# Association among cerebral glucose metabolism, mild behavioral impairment and cognition in autosomal‐dominant Alzheimer's disease

**DOI:** 10.1002/alz70856_103502

**Published:** 2025-12-26

**Authors:** Catarina Tristão‐Pereira, Ana Y Baena, Natalia Londono, Daniel Vasquez, Jorge Alcina, Lusiana Martinez, Sergio Alvarez, Monica Vidal, David Fernando Aguillón Niño, Justin S Sanchez, Vincent Malotaux, Bing He, Averi Giudicessi, Randy Medrano, Jennifer R. Gatchel, Bernard J Hanseeuw, Yakeel T. Quiroz

**Affiliations:** ^1^ Massachusetts General Hospital, Harvard Medical School, Boston, MA, USA; ^2^ Department of Psychological & Brain Sciences, Boston University, Boston, MA, USA; ^3^ Grupo de Neurociencias de Antioquia, Facultad de Medicina, Universidad de Antioquia, Medellín, Colombia; ^4^ Grupo de Neurociencias de Antioquia, Facultad de Medicina, Universidad de Antioquia, Medellín, Antioquia, Colombia; ^5^ Grupo de Neurociencias de Antioquia, Universidad de Antioquia, Medellín, Antioquia, Colombia; ^6^ Boston University, Boston, MA, USA; ^7^ Hospital Pablo Tobón Uribe, Medellín, Antioquia, Colombia; ^8^ Hospital Pablo Tobón Uribe, Medellin, Colombia; ^9^ Grupo de Neurociencias de Antioquia, Universidad de Antioquia, Medellín, Colombia; ^10^ McLean Hospital, Belmont, MA, USA; ^11^ Institute of Neuroscience, Université Catholique de Louvain, Brussels, Belgium

## Abstract

**Background:**

Neuropsychiatric symptoms, affecting 60%‐90% of dementia patients, are increasingly recognized early manifestations of disease. Mild behavioral impairment (MBI) is an emerging construct characterized by decreased motivation, affective dysregulation, disinhibition and perceptual changes in non‐demented individuals that has shown inconsistent associations with neurodegeneration. This study investigates the relationship between MBI and cerebral glucose metabolism, a marker of neuronal and astrocytic degeneration, in autosomal‐dominant Alzheimer's disease (AD).

**Method:**

Participants from the Colombia‐Boston (COLBOS) Biomarkers Study (Table 1), including 22 Presenilin‐1 E280A mutation carriers (40.2±6.9 years, 15 females, 5 cognitively impaired) and 26 cognitively unimpaired non‐carriers (38.4±5.7 years, 18 females) completed the self‐reported MBI‐Checklist (MBI‐C). They were classified as MBI‐ (MBI‐C=0) and MBI+ (MBI‐C>0). Working memory was assessed using the Consortium to Establish a Registry for Alzheimer's Disease Word List Learning. Cerebral glucose metabolism in the precuneus was measured using ^18^F‐fluorodeoxyglucose (FDG) PET. Group comparisons were performed using the Wilcoxon rank‐sum test. In a subsample with amyloid (^11^C‐Pittsburgh compound B) and tau (^18^F‐flortaucipir) PET, a mediation analysis tested whether the association between MBI and FDG uptake was mediated by AD pathology (latent variable defined as amyloid and tau).

**Result:**

The prevalence of MBI positivity was higher in carriers (68%), compared to non‐carriers (35%) (*p* = 0.041). Groups did not differ in working memory (*p* = 0.060), although MBI+ carriers tended to have lower performance. Among the whole sample, MBI+ participants had lower precuneus FDG uptake (*p* = 0.019) than MBI‐. This difference was mainly driven by MBI+ carriers, which had lower FDG uptake than MBI‐ carriers (*p* = 0.026), while no differences were found between MBI groups among non‐carriers. The association between MBI and FDG uptake was partially (70%) mediated by AD pathology (indirect effect: b=‐0.600, *p* = 0.011).

**Conclusion:**

Mutation carriers with MBI exhibited hypometabolism in an early AD‐related region, partially explained by AD pathology; however, no memory performance differences were observed. These findings suggest that MBI may serve as a risk factor for disease progression, with neuropsychiatric symptoms potentially preceding both neurodegeneration and cognitive impairment. Expanding the sample size will be essential to strengthen the evidence and further investigate MBI as a promising marker for identifying high‐risk individuals who may benefit from prevention strategies.

